# Evolutionary origin of gastrulation: insights from sponge development

**DOI:** 10.1186/1741-7007-12-26

**Published:** 2014-03-28

**Authors:** Nagayasu Nakanishi, Shunsuke Sogabe, Bernard M Degnan

**Affiliations:** 1 School of Biological Sciences, University of Queensland, Brisbane, QLD 4072, Australia

**Keywords:** Evolution, Gastrulation, Germ layers, Sponge development, *GATA*

## Abstract

**Background:**

The evolutionary origin of gastrulation—defined as a morphogenetic event that leads to the establishment of germ layers—remains a vexing question. Central to this debate is the evolutionary relationship between the cell layers of sponges (poriferans) and eumetazoan germ layers. Despite considerable attention, it remains unclear whether sponge cell layers undergo progressive fate determination akin to eumetazoan primary germ layer formation during gastrulation.

**Results:**

Here we show by cell-labelling experiments in the demosponge *Amphimedon queenslandica* that the cell layers established during embryogenesis have no relationship to the cell layers of the juvenile. In addition, juvenile epithelial cells can transdifferentiate into a range of cell types and move between cell layers. Despite the apparent lack of cell layer and fate determination and stability in this sponge, the transcription factor *GATA*, a highly conserved eumetazoan endomesodermal marker, is expressed consistently in the inner layer of *A. queenslandica* larvae and juveniles.

**Conclusions:**

Our results are compatible with sponge cell layers not undergoing progressive fate determination and thus not being homologous to eumetazoan germ layers. Nonetheless, the expression of *GATA* in the sponge inner cell layer suggests a shared ancestry with the eumetazoan endomesoderm, and that the ancestral role of *GATA* in specifying internalised cells may antedate the origin of germ layers. Together, these results support germ layers and gastrulation evolving early in eumetazoan evolution from pre-existing developmental programs used for the simple patterning of cells in the first multicellular animals.

## Background

It has been long argued and widely accepted that early metazoan evolution included the progressive addition and elaboration of cell layers, with the first animals being little more than a small sphere of epithelial cells. The addition of a second inner layer is thought to have followed, resulting in the so-called ‘gastraea’ that possessed an ectoderm and endoderm. A middle mesodermal layer is proposed to have evolved last [1,2]. It has been noted that this progression is reflected in contemporary early branching animals, with sponges and placozoans being interpreted as having one, two or no germ layers, cnidarians and ctenophores possessing two or possibly three germ layers and bilaterians having three or possibly four [2,3]. The generation of two or more germ layers by gastrulation and the consistency of the fate of these layers unites the Eumetazoa (though exceptions exist; for example, vertebrate tail-bud mesoderm [2]). Further, comparative developmental genetic evidence supports the homology of the endomesoderm [4] and ectodermal neurogenesis [5] across eumetazoans. However, it remains controversial whether the cell layers in sponges are homologous to eumetazoan germ layers and whether sponges undergo gastrulation.

**Figure 1:**
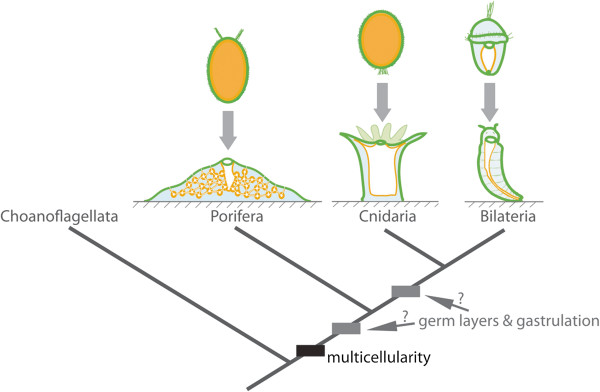
**Evolution of germ layers and gastrulation.** Animal multicellularity, with the larval and juvenile/adult body plans of extant metazoans possessing multiple cell layers, is depicted above. The arrow represents metamorphosis from larval to juvenile/adult forms. Cell layers are coloured: orange, inner layer; green, outer layer; and blue, middle layer. In the case of cnidarians and bilaterians, these correspond to endoderm, ectoderm and mesoderm (bilaterians only), respectively. The phylogenetic relationship of these clades and the sister group to metazoans, the choanoflagellates, is shown below, along with the evolutionary origin of multicellularity. The origin of germ layers and gastrulation is debatable, occurring either before or after the divergence of sponges and eumetazoans.

Sponges consist of four classes - Demospongiae, Calcarea, Homoscleromorpha and Hexactinellida [6,7] - of simple animals that share a common body organisation. As juveniles and adults, they are sessile, tri-layered animals that lack true muscles and nerves. Sponges possess an internal network of canals and ciliated choanocyte chambers lined with epithelial cells, primarily endopinacocytes and choanocytes, and are separated from the external environment by another epithelial layer, the exopinacoderm (Figure 1; hexactinellids are constructed as a syncytium). Sandwiched between these epithelial layers is the collagenous mesohyl, which is enriched with multiple types of amoebocytes, including the pluripotent stem cell type, the archeocyte (Figure 1). Water currents created by ciliary beating in choanocyte chambers enable most of the physiological requirements of sponges, including feeding, respiration, excretion and reproduction. This juvenile/adult body plan is the outcome of the dramatic reorganisation of the radially-symmetrical, bi- or trilayered larva at metamorphosis [8].

Sponges utilise recognisable gastrulation-like morphogenetic movements during embryogenesis (for example, delamination, ingression, egression and invagination) and metamorphosis (for example, epithelial-mesenchyme transitions, EMT) (summarised in [8,9]). In marine haplosclerid demosponges, for instance, morphological evidence suggests that early cleavage leads to the formation of a stereoblastula, which then delaminates to form a multilayered parenchymella larva (for example, *Amphimedon queenslandica*[10]). In homoscleromorphs (for example, *Oscarella* spp*.*), multipolar egression of a stereoblastula generates a single-layered cinctoblastula larva [11], while in a hexactinillid (for example, *Oopsacas minuta*), cellular delamination of a coeloblastula gives rise to the trichimella larva [12,13]. Interestingly, the pattern of embryogenesis can be polymorphic. In a halisarcid demosponge (for example, *Halisarca dujardini*), multipolar ingression and/or invagination of a coeloblastula or a stereoblastula forms a multilayered parenchymella or disphaerula (‘a hollow sphere within a sphere’) larva [14,15]. Epithelial-mesenchymal transitions are common at metamorphosis in diverse homoscleromorphs, calcareans and demosponges (reviewed in [8]).

Despite the occurrence of gastrulation-like morphogenetic processes in sponges, it is still unclear how sponge development relates to eumetazoan gastrulation, which is defined as a morphogenetic event that leads to the establishment of germ layers. Some authors consider cell movements that generate multi-layered embryos during sponge embryogenesis gastrulation (for example, [12,16]), although some argue that gastrulation occurs during sponge metamorphosis when an adult internal layer develops by EMT and transdifferentiation of larval ciliated epithelial cells (for example, [17]). Yet it remains unclear whether sponge cell layers display the defining characteristic of eumetazoan gastrulation. This uncertainty led others to question the existence of germ layers, and hence gastrulation, in sponges (for example, [18]).

Here, we show by cell-labelling experiments in the demosponge *A. queenslandica* that the cell layers established during embryogenesis lack fixed identities and do not directly give rise to the cell layers of the juvenile. In addition, inner epithelial choanocytes in the juvenile can alter their fate by transdifferentiating into a range of cell types, including cells of the outer epithelium. Thus, sponge cell layers lack fate determination and stability. However, we find that expression of the sponge orthologue to the highly conserved eumetazoan endomesodermal marker *GATA*[4] is consistently restricted to the inner layer of larvae and juveniles, suggesting a deep shared ancestry between the sponge internal cell layer and the eumetazoan endomesoderm.

## Results and discussion

Germ layers by definition exhibit restricted cell fate [2,3]. Thus, to address whether sponge embryonic and early juvenile cell layers are germ layers, we employed cell-labelling techniques in the demosponge *A. queenslandica* to examine the fate of larval epithelial and internal cells at metamorphosis, as well as the stability and fate of differentiated choanocytes in newly established juvenile tissues at the so-called ‘rhagon’ stage (staging described in Additional file 1: Figure S1).

The swimming *A. queenslandica* larva metamorphoses into a feeding juvenile with choanocyte chambers within three days of attaching its anterior end onto a natural substratum (crustose coralline algae) at 25°C. Within hours of settling, the three-layered, bullet-shaped larva transforms into an encrusting mat. A previous cell-labelling study in *A. queenslandica* indicated that external larval cells transdifferentiated into internal choanocytes in juveniles [10]. However, it was not determined whether all cell types found on the larval exterior are transdifferentiating at metamorphosis, and whether the larval epithelial cells differentiate directly into choanocytes or indirectly via an intermediate cell type. In other sponges, the larval epithelial layer has been reported to shed entirely [19], be phagocytised by archeocytes [20-22], differentiate into choanocytes through a non-ciliated amoebocyte intermediate [23-25], or directly differentiate into choanocytes without loss of cilia [26] (reviewed in [8]). Using the terminal deoxynucleotidyl transferase dUTP nick end-labelling (TUNEL) assay for detecting apoptotic DNA fragmentation, we demonstrate that a large proportion of *A. queenslandica* larval epithelial cells undergo programmed cell death shortly after the onset of metamorphosis [see Additional file 2: Figure S2]. TUNEL-labelled nuclear fragments can be observed readily in the metamorphosing animal, often as phagocytised bodies within archeocytes [see Additional file 2: Figure S2].

**Figure 2:**
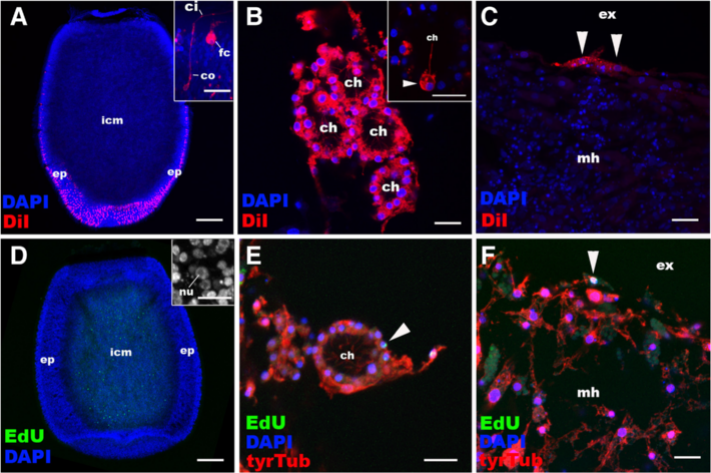
**Cell labelling and lineage-tracing through metamorphosis in *Amphimedon queenslandica. *A**, **D**: Distributions of labelled cells in free-swimming larvae. Anterior (swimming axis) is to the bottom. **B**, **C**, **E**, **F**: Descendants of the labelled cells in juveniles. Nuclei are stained with DAPI. In **E** and **F**, the juveniles are labelled with an antibody against tyrosinated tubulin (tyrTub). **A**: Longitudinal confocal sections through the centre of a free-swimming larva incubated with CM-DiI, showing strong labelling in ciliated epidermal cell types, the columnar epithelial cell (co) and the flask cell (fc) (inset), with little labelling in inner cell mass (icm). **B**: Choanocytes in chambers (ch). In some cases, a subset of choanocytes is CM-DiI-labelled in a single choanocyte chamber (arrowhead in inset), suggesting that multiple precursor cells can be involved in development of a single chamber. **C**: Labelled exopinacocytes (arrowheads). **D**: A confocal longitudinal section through the centre of a free-swimming larva pulse-labelled with EdU. Note that the labelled cells localise in the inner cell mass (icm) and are likely to be proliferating archeocytes with characteristic large nucleoli (nu) [see Additional file 3: Figure S3]. **E**: An EdU-positive choanocyte in the chamber (arrowhead). **F**: An EdU-positive exopinacocyte (arrowhead). Other abbreviations: ep, outer layer epithelium; ci, cilium; ex external environment; mh, mesohyl. Scale bars: 100 μm **(A, D)**, 10 μm (**B**, **C**, **E**, **F**, inset in **D**), 5 μm (inset in **A**). DAPI, 4',6-diamidino-2-phenylindole; EdU, 5-ethynyl-2′-deoxyuridine.

To study the fate of non-apoptosing larval epithelial cells, the outer layer was labelled with the plasma-membrane dye CM-DiI. We assume that the cell lineage will be indicated by retention of this label in their plasma membrane, while the plasma membrane of the cells that phagocytised apoptotic fragments of larval epithelial origin will not be labelled. An overnight incubation of free-swimming *A. queenslandica* larvae in seawater containing 10 μM CM-DiI strongly labelled the plasma membrane of ciliated epithelial cell types, the columnar epithelial cell and the flask cell (Figure 2A). During metamorphosis, these labelled cells internalised, resorbing their cilia and differentiating into archeocytes, which migrated throughout the animal [see Additional file 3: Figure S3]. Within two to three days, a number of juvenile cell types were labelled, including choanocytes and exopinacocytes (Figure 2B, C), suggesting that these differentiated from pluripotent archeocytes, which were originally derived from the larval epithelium. Therefore, at metamorphosis ciliated epidermal cells of the larvae can either undergo apoptosis or transdifferentiate, via an archeocyte intermediate, into a range of juvenile cell types that are found in the inner, outer or middle layer of the juvenile.

Using the thymidine analog, 5-ethynyl-2′-deoxyuridine (EdU), to label the nuclei of proliferating cells, we found that only amoeboid cells localised to the inner cell mass, identified here as archeocytes [see Additional file 4: Figure S4], and no other cell types, continue to proliferate in the swimming larva (Figure 2D). Taking advantage of this observation we followed the fate of these EdU-labelled cells during metamorphosis. We found that these archeocytes of the larval inner cell mass origin also gave rise to choanocytes and exopinacocytes during metamorphosis (Figure 2E, F). Together, these observations indicate that a range of larval cell types can generate the diversity of cell types present in the juvenile and that there is no correspondence between the cell layers established during embryogenesis and those produced at metamorphosis.

**Figure 3:**
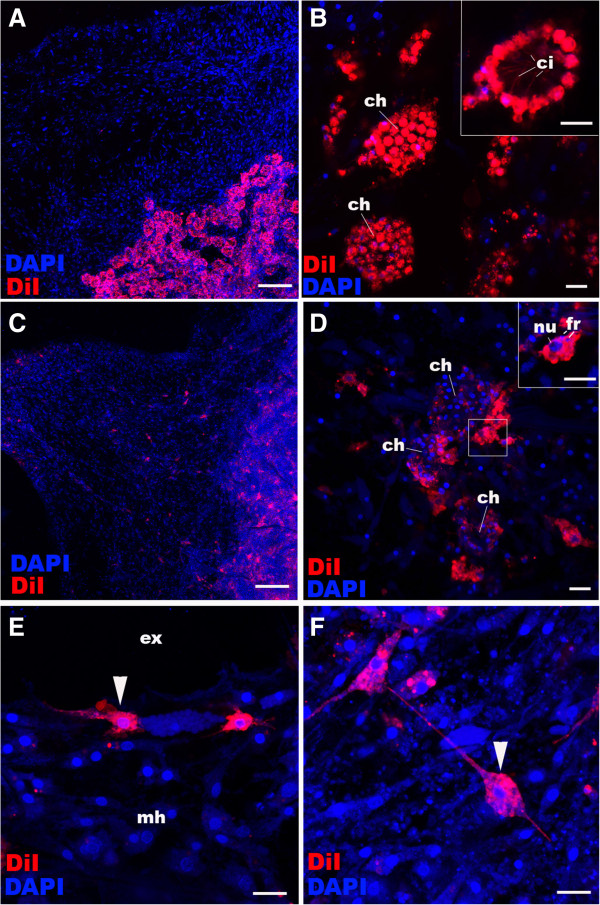
**Choanocyte labelling and lineage-tracing in juvenile *Amphimedon queenslandica. *A**, **B**: Choanocytes in chambers (ch) in juveniles are labelled with CM-DiI; note strong plasma membrane labelling in choanocytes (inset). **C-F**: Descendants of juveniles whose chambers were labelled with CM-DiI seven days earlier (as in **A**). Multiple other juvenile cell types are now labelled and spread through the sponge body. Note in **(D)** the limited number of CM-DiI-labelled choanocytes remaining in the choanocyte chambers (ch) seven days after labelling and the presence of labelled archeocytes with large nuclei (nu; inset), consistent with transdifferentiation of choanocytes into archeocytes. Note the presence of nuclear fragments (fr) presumably resulting from phagocytosis that has occurred during de-differentiation [see Additional file 5: Figure S5]. Arrowheads in **E** and **F** show a labelled exopinacocyte and a sclerocyte, respectively, indicating these cell types can also be produced from de-differentiating choanocytes. Nuclei are stained with DAPI. Scale bars: 100 μm **(A, C)**, 10 μm **(B, D-F)**. DAPI, 4',6-diamidino-2-phenylindole.

Sponge metamorphosis has been regarded by some as gastrulation (summarised in [8,17]). As observed here and in previous studies, morphogenetic processes operational during sponge metamorphosis occur also during eumetazoan gastrulation (for example, EMT; reviewed in [8,27]). Therefore, we sought to determine if cell layers established at the end of *A. queenslandica* metamorphosis are developmentally determined. To test this, we labelled choanocytes that form internal choanocyte chambers and a small number of archeocytes surrounding choanocyte chambers by incubating juveniles with CM-DiI (Figure 3A, B). Within one week, the majority of the labelled choanocytes had undergone EMT and dedifferentiated into archeocytes (Figure 3C, D; Additional file 5: Figure S5), of which some had migrated away from the chambers and differentiated into other cell types including exopinacocytes (Figure 3E) and sclerocytes (Figure 3F). Thus, inner epithelial choanocytes are capable of transdifferentiating into outer epithelial cells via an archeocyte intermediate, demonstrating the lack of cell fate determination.

The lack of fate determination in cell layers in *A. queenslandica* is consistent with this demosponge lacking germ layers as classically defined. In light of this finding, genes shown to be instrumental in eumetazoan ectoderm or endomesoderm formation may not be expected to be present in demosponges. Indeed, of the genes previously implicated in the differentiation of the endomesoderm across Cnidaria and Bilateria, namely *twist, snail, forkhead* and *GATA*[4], only *GATA* was identified in the genome of the demosponge *A. queenslandica*[28] [see Additional file 6: Figure S6]. Also, amongst the 51 transcription factor- and signalling molecule-encoding genes shown to be ‘endomesodermally’ expressed in the cnidarian *Nematostella vectensis*[29], only nine orthologues are present in the *A. queenslandica* genome [see Additional file 7: Table S1].

**Figure 4:**
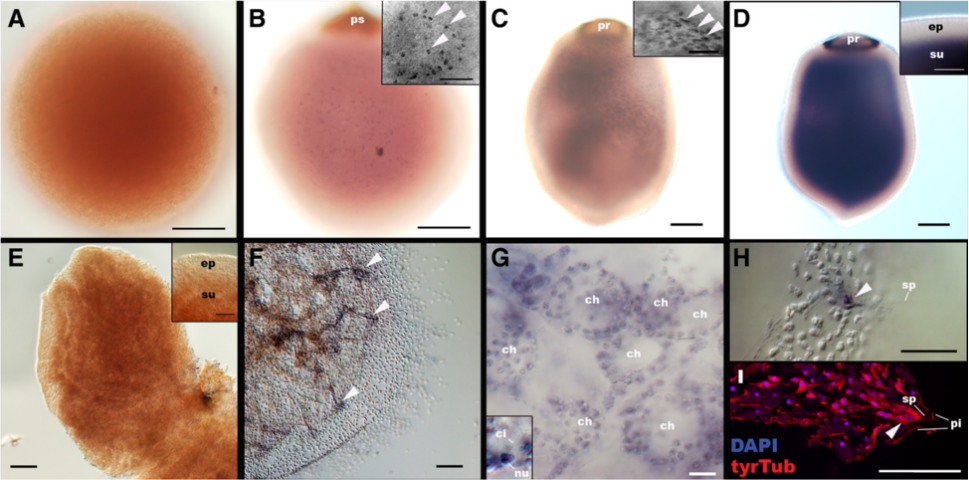
***GATA *mRNA expression during development in *A. queenslandica*.** Late blastulae (**A**; *sensu* Leys and Degnan [10]), mid-stage embryos with a pigment spot (ps) **(B)**, late-stage embryos with a pigment ring (pr) **(C)**, a free-swimming larva **(D)**, a settlement stage postlarva **(E)**, and a juvenile **(F-I)** were labelled with the antisense riboprobe *AqGATA*. The juvenile in I is labelled with DAPI and an anti-tyrTub antibody. In **B-D**, the specimens are viewed from the lateral side with the posterior pigmented structures (ps and pr) placed up in each panel. In **E** and **F**, the specimens are viewed from the top of postlarva. Medial optical sections exposing the internally localised subepithelial cells are shown. Insets in **B** and **C** show *GATA* expression in individual subepithelial cells (arrowheads), and an inset in **D** shows the demarcation between the *GATA*-negative epidermal layer (ep) and the strongly *GATA*-positive subepithelial domain (su). An inset in **E** shows the downregulation of *GATA* expression in the subepithelial domain (su) relative to the outer layer epithelium at settlement. Arrowheads in **F** indicate the internal spicule-forming tent-pole-like structures in the juvenile that strongly express *GATA*. In **G**, an inset shows a *GATA-*expressing choanocyte with a cilium (ci) and a basal nucleus (nu). An arrowhead in **H** shows a cluster of *GATA*-expressing spicule (sp)-forming cells at the apex of the tent-pole-like structures shown in **F**; note in **I** that their cell bodies (arrowhead) are situated basal to the pinacoderm (pi). Scale bars: 100 μm **(A-F)**, 50 μm (**H**, **I**, and insets in **B-E**), 10 μm **(G)**. DAPI, 4',6-diamidino-2-phenylindole.

*A. queenslandica GATA* transcripts are localised to a substantial fraction of internal cells of late-stage embryos, free-swimming larvae (Figure 4B-D) and juveniles (Figure 4F-I), but not detectable in outer layer epithelial cells at any developmental stage, similar to predominantly internal (that is, endomesodermal) expression of bilaterian *GATA456*[30-32] and cnidarian *GATA*[4]. Thus, although this study argues against the existence of true germ layers in demosponges*,* the striking parallel in *GATA* expression patterns suggests a common ancestry between the sponge inner cell layer and the eumetazoan endomesoderm.

*GATA* genes are present in other early branching taxa, including placozoans, ctenophores and the other classes of sponges [33-35], although *A. queenslandica* is the only species for which there currently are published expression patterns. Understanding the evolution of germ layers will be assisted by the analysis of the evolution and expression of genes conserved in bilaterian mesoderm specification and formation, along with further analysis of developmental processes in these taxa. For example, recent analysis of the genome of the ctenophore *Mnemiopsis leidyi* failed to reveal many ‘mesodermal’ genes despite it appearing to have genuine endomesoderm [34]*,* and it is known that cell movements from one embryonic layer to another occur during metamorphosis in some medusozoan cnidarians [36,37]. These approaches, along with resolving the exact order in which these early-branching metazoans diverged from the lineage leading to bilaterians (that is, whether sponges or ctenophores are the sister group to all other animals and whether sponges are monophyletic [34,38]), provide an opportunity to reconstruct the evolution of germ layers and gastrulation.

## Conclusions

Comparison of demosponge and eumetazoan development and body plans minimally provides insights into their last common ancestor, which appears to have existed some 800 million years ago [39]. Although the possibility that their last common ancestor possessed germ layers and these were lost in the demosponge lineage cannot be excluded, the lack of developmental commitment of larval and juvenile cell types in *A. queenslandica* and homology of internal layers across a demosponge and eumetazoans are consistent with eumetazoan germ layers and gastrulation evolving after the divergence of demosponge and eumetazoan lineages. Thus, we propose that ancestrally metazoans consisted of multiple cell layers whose fate was labile. The evolution of the gene regulatory network endowing progressive determination of cell layers resulted in the emergence of primary germ layers and mechanisms to segregate these layers in eumetazoans (that is, gastrulation). The origin of the majority of genes that have conserved roles in eumetazoan gastrulation and germ layer determination evolved after the divergence of demosponge and eumetazoan lineages (for example, *Fkh*, *Twist*, *Snail*), giving credence to this supposition. The conserved expression of *GATA* in cells located inside the body of all metazoans, along with conserved differential expression of Wnt and TGF-β ligands along demosponge and eumetazoan body axes [40], suggests that these genes were essential in providing critical positional information to cells in the first multicellular metazoans. These, along with eumetazoan-specific genes, later became essential components of the specification and determination of eumetazoan germ layers.

## Methods

### Animal collection

Adult *A. queenslandica* were collected at the Heron Island Research Station, Queensland, Australia. Larvae were collected upon release from adults and metamorphosing individuals were obtained by allowing larvae to settle and metamorphose on a plastic petri dish as previously described [41].

### Cell lineage tracing with CM-DiI

The lipophilic tracer CM-DiI (Molecular Probes, C7000, Mulgrave, Victoria, AUS) was used to label ciliated epithelial cells in larvae and choanocytes in the juveniles. Larvae or juveniles were incubated in seawater with 10 μM CM-DiI. To differentially label the plasma membrane of ciliated epithelial cells, larvae were incubated for 12 hours. Incubation for one hour was sufficient to differentially label the majority of choanocytes in juveniles. Little labelling occurred in internal cells in larvae or juveniles. The labelled specimens were rinsed in fresh seawater several times and then allowed to develop further. Specimens were fixed as described below at various stages of development. Nuclei were labelled with the fluorescent dye 4',6-diamidino-2-phenylindole (DAPI; 1:1,000, Molecular Probes), and the specimens were mounted in ProlongGold antifade reagent (Molecular Probes).

### EdU pulse-and-chase experiments

Larvae or juveniles were incubated in seawater containing 200 μM of the thymidine analogue, EdU (Click-iT EdU AlexaFluor 488 cell proliferation kit, C10337, Molecular Probes), for 16 to 18 hours to label nuclei of proliferating larval archeocytes, and for three hours to label S-phase nuclei in the juveniles. Following washes in fresh seawater, the juveniles were immediately fixed. The larvae with labelled archeocytes were allowed to metamorphose into the juvenile stage, which were then fixed. Fixed specimens underwent immunohistochemistry as described below. Following the immunohistochemistry procedure, fluorescent labelling of incorporated EdU was conducted according to the manufacture’s recommendations prior to DAPI labelling.

### Immunohistochemistry, TUNEL and confocal microscopy

Larvae, postlarvae and juveniles were fixed as previously described [42]. Fixed specimens were rehydrated and washed in PBST (phosphate-buffered saline + 0.1% Tween20) and blocked with 1% bovine serum albumin (BSA) in PBST. Primary antibodies were incubated with the specimens in PBST. We used antibodies against phosphorylated histone H3 (phospho S10) (rabbit, 1:500, Abcam ab5176, Cambridge, MA, USA) and tyrosinated-∂-tubulin (mouse, 1:500, Sigma T9028, Castle Hill, NSW, AUS) overnight at 4°C. Following washes in PBST and blocking in 1% BSA in PBST, secondary antibodies were incubated with the specimens; we used AlexaFluor 568 (anti-rabbit or -mouse. 1:200, Molecular Probes) and AlexaFluor 647 (anti-rabbit or -mouse, 1:200, Molecular Probes). Filamentous actin was labelled using AlexaFluor 488-conjugated phallacidin (1:25, Molecular Probes). Specimens were incubated with phallacidin together with secondary antibodies. After washes in PBST, the TUNEL assay was performed by using an In Situ Cell Death Detection Kit (TMR red cat no. 1684795, Roche, Indianapolis, IN, USA) following the manufacturer’s protocol. Nuclei were labelled with DAPI and the specimens were mounted in ProlongGold antifade reagent as described above. Images were recorded using a Zeiss LSM 510 META Confocal Microscope and confocal stacks were viewed using ImageJ.

### Gene isolation and whole-mount *in situ* hybridization

*AqGATA* gene fragments were amplified with gene specific primers, by using complimentary DNA from mixed developmental stages as a PCR template. Gene specific primers were as follows: F1, ATGGAGAAAGCAGACGACGCTATGC; F2, CTACCAAGACAGTTTTGTGG; R1, CTACATCAATTGCTGTGGTTGCATGG and R2, ACTTTGACTTCTTTGACTCG. Primers F2 and R1 were used to generate a 776 base template for a riboprobe synthesis. Riboprobe synthesis and *in situ* hybridization were conducted as described previously [42]. An antisense digoxigenin-labeled riboprobe was hybridised at a final concentration of 1 ng/μl.

### Transmission electron microscopy

Free-swimming larvae were processed for transmission electron microscopy (TEM) by using the high-pressure freezing (HPF) technique. Procedures followed the previously established HPF protocol for shark retinal tissues [43] with the following modifications: 20% BSA in artificial seawater was used as a cryoprotectant instead of the teleost ringer/0.7% agarose. In addition, 1% OsO_4_ was used in the freeze substitution cocktail. Longitudinal ultrathin sections were prepared and analysed.

## Competing interests

The authors declare that they have no competing interests.

## Authors’ contributions

NN and SS carried out the experiments and microscopy. NN and BMD conceived of the study and wrote the manuscript. All authors read and approved the final manuscript.

## Supplementary Material

Additional file 1: Figure S1 Stages of development during metamorphosis in *A. queenslandica*. **A**: Free-swimming larva stage; the (parenchymella) larva is egg-shaped and swims with the pigmented ring and associated bundles of long cilia directed posteriorly [10]. The anterior is down. In **B-F**, metamorphosing postlarvae are viewed from the top. **B**: Settlement stage. The anterior region of the larva is attached to the substrate, onto which the larva flattens. **C**: Mat-formation postlarval stage, cells of the metamorphosing postlarva migrate laterally on the substrate to form a mat-like structure. Note that former posterior-ring pigmentation (pi) is still visible but is disappearing. **D**: Chamber-formation postlarval stage, with developing canals (ca) lined by choanocyte chambers (ch) and endopinacoderm. Note the aquiferous system composed of canals lined by choanocytes and endopinacocytes becomes evident. **E**: Tent-pole-formation postlarval stage, the exopinacotes covering the outer surface of the metamorphosing postlarva are lifted upwards by formation of tent-pole-like structures consisting of vertically oriented clusters of spicules and associated cells. Arrowheads show the internal tent-pole-like structures, visible here as clustering of cells. **F**: Juvenile (rhagon) stage with an osculum (os), marking the establishment of the functional aquiferous system. Abbreviations: lc long cilia; pr pigment ring; cc cuboidal cells. Scale bar: 100 μm (**A**, inset in **F**), 1 mm **(B-F)**. Click here for file

Additional file 2: Figure S2 Apoptosis, loss of epithelial integrity and phagocytosis at early metamorphosis in A. queenslandica. **A-C**: Free-swimming larva. **D-F**: Settled postlarva, ≤7 hours post-settlement. **G**: Settled postlarva, ≤12 hours post-settlement. **H**: Mat-formation postlarval stage. DAPI is used to label DNA. In **A**, **B**, **D**, **E** and **G**, DNA fragmentation is detected by TUNEL assays (TUNEL). In **C**, **F** and **G**, specimens are labelled with an anti-Tyrosinated-Tubulin (tyrTub). **A**: A confocal longitudinal section through the larva; anterior is down. A boxed region in **A** is shown in **B**; note that TUNEL-positive fragmented DNA (arrowheads) localises within the most anterior domain where cuboidal cells reside. **C**: A confocal section through the outer layer epithelium; note that the epithelium consists of a continuous sheet of cells with apical cilia (ci) and basal, typically anucleolated, nuclei (nu). **D**: Confocal medial sections viewed from the top of the metamorphosing postlarva. A single section through the outer layer epithelium in the boxed region in **D** is shown in **E**. **F**: A confocal section showing the surface view of the distintegrating outer layer epithelium; note the breakup into smaller, tightly packed cells with anucleolated nuclei (the fragmented epithelium; fr). **G**: A confocal section of an outer-most region of the metamorphosing postlarva; note the lack of an epithelium, and the presence of phagocytised TUNEL-positive apoptotic bodies (arrowheads). **H**: A confocal section through an archeocyte in a mat-formation-stage postlarva labelled with DAPI and an anti-tyrTub, in which larval archeocytes and ciliated epidermal cells were simultaneously labelled (with EdU and CM-DiI, respectively) and followed through metamorphosis. Note intracellular CM-DiI-positive cellular fragments containing DAPI-positive nuclear materials (arrowhead), the presumptive apoptotic bodies, in an EdU-positive archeocyte of larval origin. Abbreviations: ep outer layer epithelium; icm inner cell mass. Scale bars: 100 μm **(A, D, E)**, 10 μm **(B, C, F-H)**. Click here for file

Additional file 3: Figure S3 Larval ciliated epithelial cells lose cilia and transdifferentiate into choanocytes via an archeocyte intermediate during metamorphosis in *A. queenslandica.* Confocal sections through a flask cell (fc) at the free-swimming larva stage **(A)** and a transdifferentiating flask cell at the settlement stage labelled with CM-DiI **(B)**. Note the loss of apical structures including the cilium (ci). **C**: Confocal sections viewed from the top at settlement, showing that many DiI-labelled cells are internalised (arrowheads). **D**: Confocal sections viewed from the top during metamorphosis; note that DiI-labelled cells have differentiated into archeocytes with characteristic large nucleoli (nu; inset magnified from the boxed cells). **E**: Confocal sections of individual archeocytes of the larval epidermal origin undergoing further differentiation at the chamber-formation stage; an arrowhead shows a differentiating choanocyte with a cilium (ci in inset) whose cell body has yet to complete cytokinesis within the mother archeocyte (ar). **F**: Confocal sections of a multi-nucleated archeocyte-choanocyte intermediate at the chamber-formation stage. Differentiating choanocytes with cilia (ci) are evident (arrowheads). Abbreviations: ap apical non-ciliary process; ba basal process; co columnar epithelial cell. Scale bar: 10 μm (**A**, **B**, inset in **D**, **E**, **F**), 100 μm **(C, D)**. Click here for file

Additional file 4: Figure S4 EdU is incorporated into proliferating archeocytes in the inner cell mass in *A. queenlandica* larvae. **A, B**: Confocal sections of the inner cell mass in a free-swimming larva pulse-labelled with EdU. Nuclei are labelled with DAPI. Arrowheads show EdU-positive nuclei. The boxed region in **B** is magnified in an inset. Note that the EdU-positive nuclei are 3.5 to 4.0 μm in diameter and contain prominent nucleoli (nu), as observed in larval archeocytes in the inner cell mass in another demosponge *Haliclona tubifera*[44]. **C**: A transmission electron micrograph showing prominent nucleoli (nu) in nuclei of archeocytes (ar) in the inner cell mass within an *A. queenslandica* larva. Scale bar: 10 μm **(A, B)**, 5 μm **(C)**. Click here for file

Additional file 5: Figure S5 Epithelial choanocytes can de-differentiate into archeocytes in *A. queenslandica*. Choanocytes in chambers (ch) in juveniles were labelled with CM-DiI as in Figure 3A, B and descendants of the labelled cells in juveniles one day later are shown. Some choanocytes are seen to extend basal processes (ba) laterally, engulfing neighboring choanocytes (arrowheads in **A**, **B**). Some cells appear multinucleated (arrowheads in **C**), presumably as a result of phagocytic or fusion events. Abbreviations: ch choanocyte chamber; ci cilium. Scale bar: 10 μm. Click here for file

Additional file 6: Figure S6 GATA sequence alignment and phylogenetic analyses. **A**: Partial GATA protein multiple sequence alignment showing the amino acid sites used for phylogenetic analyses. GATA genes encode zinc-finger transcription factors that bind to the (T/A (GATA) A/G) cis-regulatory motif [45] and are characterised by the presence of two zinc-finger domains (N-terminal ‘N-finger’ and C-terminal ‘C-finger’). The alignment encompasses N-finger (underlined in red) and C-finger (underlined in blue) zinc-finger domains each encoding CXNC (boxed) X_17_CNXC (boxed). **B**: GATA maximum likelihood phylogeny rooted with GATA-type sequences. *Amphimedon queenslandica* sequence is highlighted in red. No clear GATA orthologues were identified outside Metazoa, and in the genome of the ctenophore *Mnemiopsis leidyi*[46]. **C**: Unrooted GATA maximum likelihood phylogeny. Sequence alignment and phylogenetic analyses were performed on the Geneious platform (v.5.1.7). Related sequences were retrieved via the protein BLAST search using the Amphimedon GATA-like sequence as queries, from GenBank at NCBI [47], Acropora digitifera genome (Version 1.1) portal at OIST [48], Mnemiopsis genome project portal at NIH [46] and Compagen at the University of Kiel (Oscarella sequences; [49]). Sequence IDs/accession numbers are shown with the name of each sequence in **B** and **C**. Peptide sequences were aligned with MUSCLE (v3.7) [50] (default settings), and ambiguous regions were manually removed. Phylogenetic trees were reconstructed using the maximum likelihood method implemented in the PhyML program [51]. The WAG substitution model [52] was selected assuming an estimated proportion of invariant sites and four gamma-distributed rate categories to account for rate heterogeneity across sites. The gamma shape parameter was estimated directly from the data. Reliability for internal branches of maximum likelihood trees was assessed using the bootstrapping method (100 bootstrap replicates). Support values are shown at each node except when lower than 50%. The unit of the branch length is the number of substitutions per site. Click here for file

Additional file 7: Table S1 A list of the presence or absence of *A. queenslandica* homologues (orthologues and gene family members) to cnidarian *Nematostella vectensis* endomesodermal genes (listed in [29]) based on previously published data [28,53-58] and sequence similarity searches in NCBI databases (via BLASTp). Click here for file
