# Supplementary figures and images for: Evolutionary origin of gastrulation: insights from sponge development
Source: BMC Biol. 2014 Mar 28;12:26. doi: 10.1186/1741-7007-12-26 (PMC4021757; doi:10.1186/1741-7007-12-26)

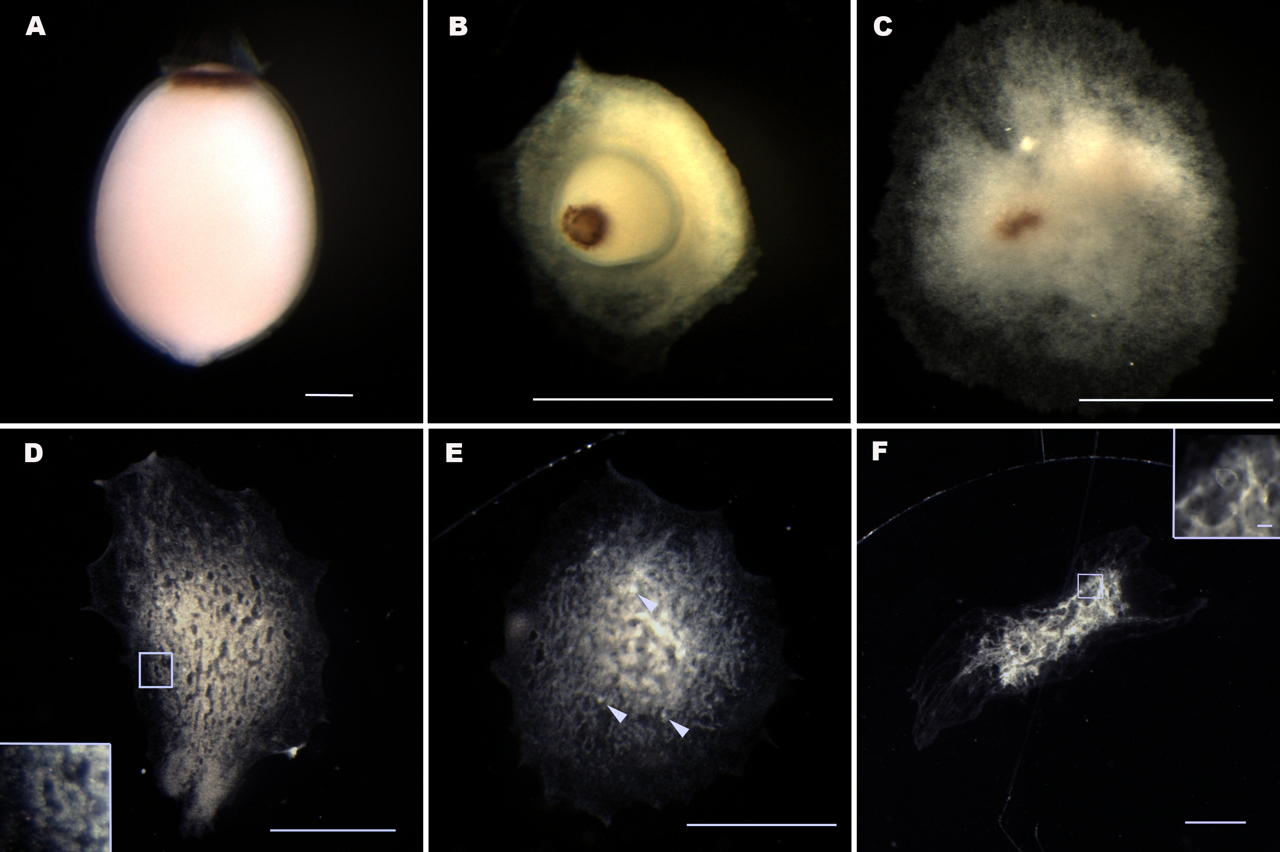

Supplement: Additional file 1: Figure S1 — Stages of development during metamorphosis in A. queenslandica. A: Free-swimming larva stage; the (parenchymella) larva is egg-shaped and swims with the pigmented ring and associated bundles of long cilia directed posteriorly [10]. The anterior is down. In B-F, metamorphosing postlarvae are viewed from the top. B: Settlement stage. The anterior region of the larva is attached to the substrate, onto which the larva flattens. C: Mat-formation postlarval stage, cells of the metamorphosing postlarva migrate laterally on the substrate to form a mat-like structure. Note that former posterior-ring pigmentation (pi) is still visible but is disappearing. D: Chamber-formation postlarval stage, with developing canals (ca) lined by choanocyte chambers (ch) and endopinacoderm. Note the aquiferous system composed of canals lined by choanocytes and endopinacocytes becomes evident. E: Tent-pole-formation postlarval stage, the exopinacotes covering the outer surface of the metamorphosing postlarva are lifted upwards by formation of tent-pole-like structures consisting of vertically oriented clusters of spicules and associated cells. Arrowheads show the internal tent-pole-like structures, visible here as clustering of cells. F: Juvenile (rhagon) stage with an osculum (os), marking the establishment of the functional aquiferous system. Abbreviations: lc long cilia; pr pigment ring; cc cuboidal cells. Scale bar: 100 μm (A, inset in F), 1 mm (B-F). [file 1741-7007-12-26-S1.tiff]

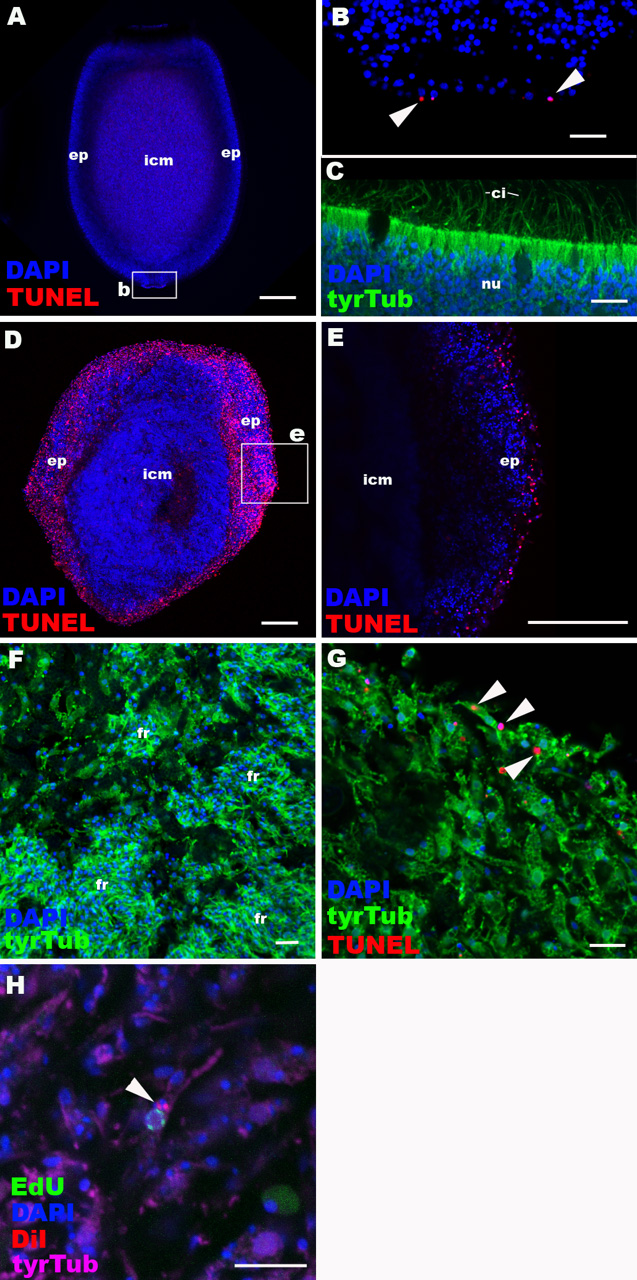

Supplement: Additional file 2: Figure S2 — Apoptosis, loss of epithelial integrity and phagocytosis at early metamorphosis in A. queenslandica. A-C: Free-swimming larva. D-F: Settled postlarva, ≤7 hours post-settlement. G: Settled postlarva, ≤12 hours post-settlement. H: Mat-formation postlarval stage. DAPI is used to label DNA. In A, B, D, E and G, DNA fragmentation is detected by TUNEL assays (TUNEL). In C, F and G, specimens are labelled with an anti-Tyrosinated-Tubulin (tyrTub). A: A confocal longitudinal section through the larva; anterior is down. A boxed region in A is shown in B; note that TUNEL-positive fragmented DNA (arrowheads) localises within the most anterior domain where cuboidal cells reside. C: A confocal section through the outer layer epithelium; note that the epithelium consists of a continuous sheet of cells with apical cilia (ci) and basal, typically anucleolated, nuclei (nu). D: Confocal medial sections viewed from the top of the metamorphosing postlarva. A single section through the outer layer epithelium in the boxed region in D is shown in E. F: A confocal section showing the surface view of the distintegrating outer layer epithelium; note the breakup into smaller, tightly packed cells with anucleolated nuclei (the fragmented epithelium; fr). G: A confocal section of an outer-most region of the metamorphosing postlarva; note the lack of an epithelium, and the presence of phagocytised TUNEL-positive apoptotic bodies (arrowheads). H: A confocal section through an archeocyte in a mat-formation-stage postlarva labelled with DAPI and an anti-tyrTub, in which larval archeocytes and ciliated epidermal cells were simultaneously labelled (with EdU and CM-DiI, respectively) and followed through metamorphosis. Note intracellular CM-DiI-positive cellular fragments containing DAPI-positive nuclear materials (arrowhead), the presumptive apoptotic bodies, in an EdU-positive archeocyte of larval origin. Abbreviations: ep outer layer epithelium; icm inner cell mass. Scale bars: 100 μm (A, D, E), 10 [file 1741-7007-12-26-S2.jpeg]

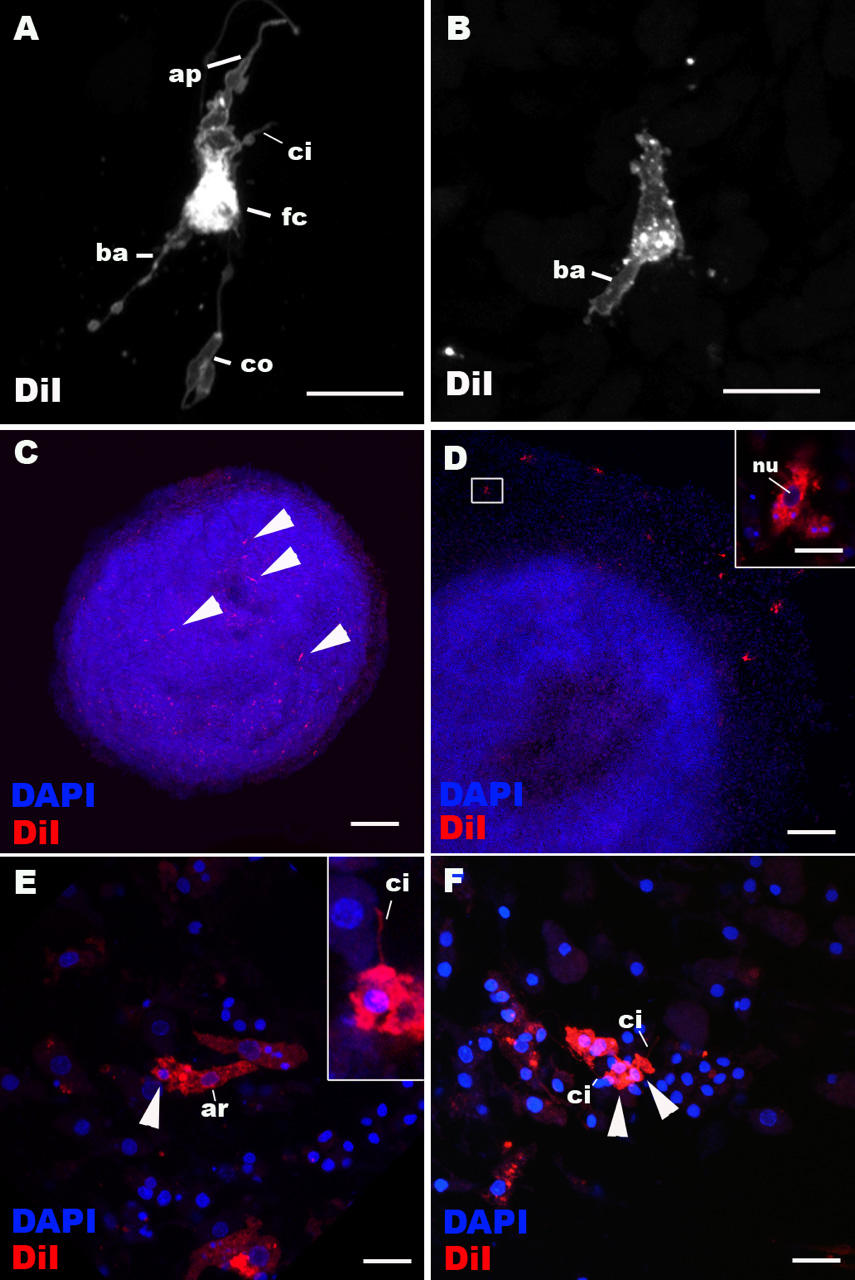

Supplement: Additional file 3: Figure S3 — Larval ciliated epithelial cells lose cilia and transdifferentiate into choanocytes via an archeocyte intermediate during metamorphosis in A. queenslandica. Confocal sections through a flask cell (fc) at the free-swimming larva stage (A) and a transdifferentiating flask cell at the settlement stage labelled with CM-DiI (B). Note the loss of apical structures including the cilium (ci). C: Confocal sections viewed from the top at settlement, showing that many DiI-labelled cells are internalised (arrowheads). D: Confocal sections viewed from the top during metamorphosis; note that DiI-labelled cells have differentiated into archeocytes with characteristic large nucleoli (nu; inset magnified from the boxed cells). E: Confocal sections of individual archeocytes of the larval epidermal origin undergoing further differentiation at the chamber-formation stage; an arrowhead shows a differentiating choanocyte with a cilium (ci in inset) whose cell body has yet to complete cytokinesis within the mother archeocyte (ar). F: Confocal sections of a multi-nucleated archeocyte-choanocyte intermediate at the chamber-formation stage. Differentiating choanocytes with cilia (ci) are evident (arrowheads). Abbreviations: ap apical non-ciliary process; ba basal process; co columnar epithelial cell. Scale bar: 10 μm (A, B, inset in D, E, F), 100 μm (C, D). [file 1741-7007-12-26-S3.jpeg]

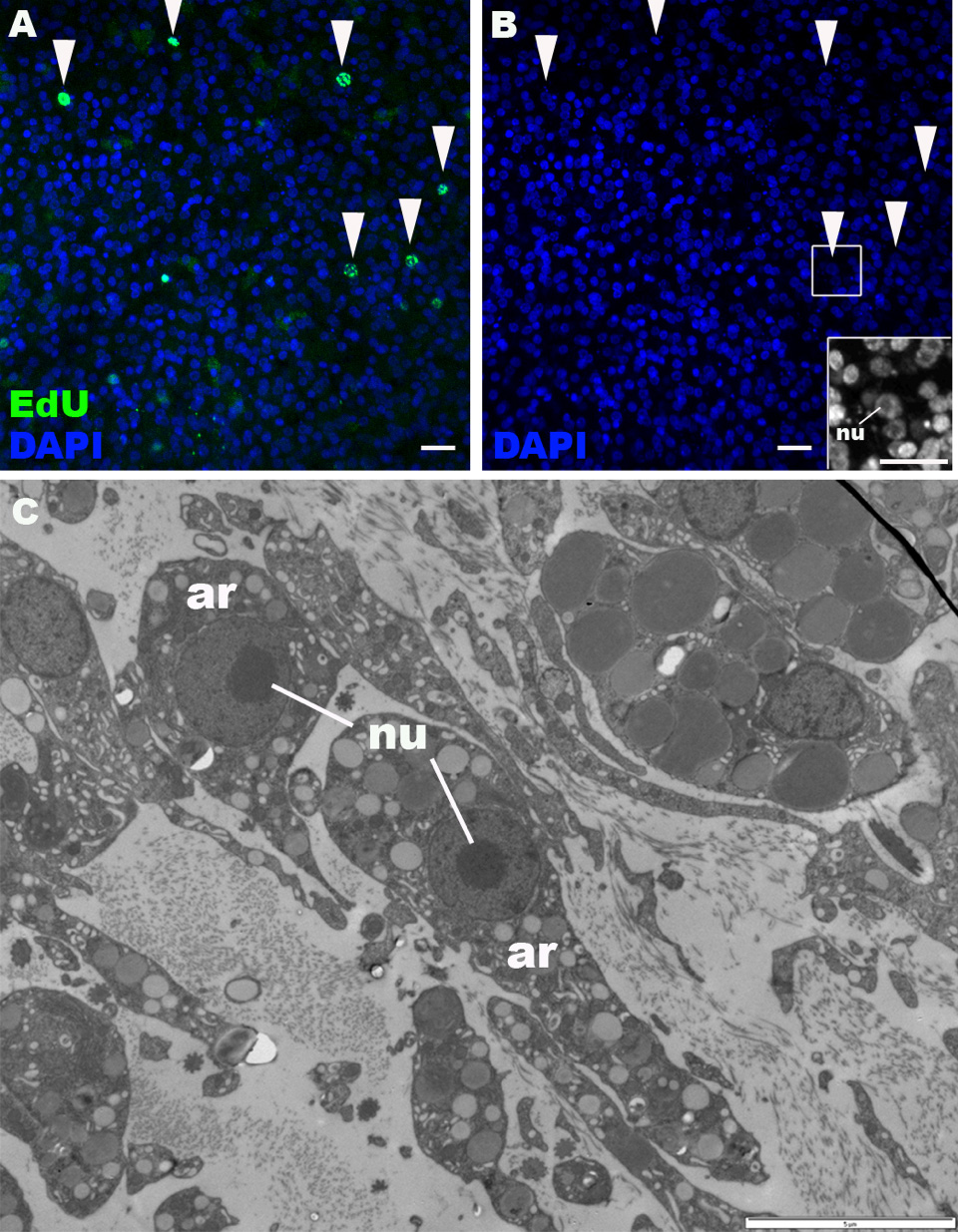

Supplement: Additional file 4: Figure S4 — EdU is incorporated into proliferating archeocytes in the inner cell mass in A. queenlandica larvae. A, B: Confocal sections of the inner cell mass in a free-swimming larva pulse-labelled with EdU. Nuclei are labelled with DAPI. Arrowheads show EdU-positive nuclei. The boxed region in B is magnified in an inset. Note that the EdU-positive nuclei are 3.5 to 4.0 μm in diameter and contain prominent nucleoli (nu), as observed in larval archeocytes in the inner cell mass in another demosponge Haliclona tubifera[44]. C: A transmission electron micrograph showing prominent nucleoli (nu) in nuclei of archeocytes (ar) in the inner cell mass within an A. queenslandica larva. Scale bar: 10 μm (A, B), 5 μm (C). [file 1741-7007-12-26-S4.jpeg]

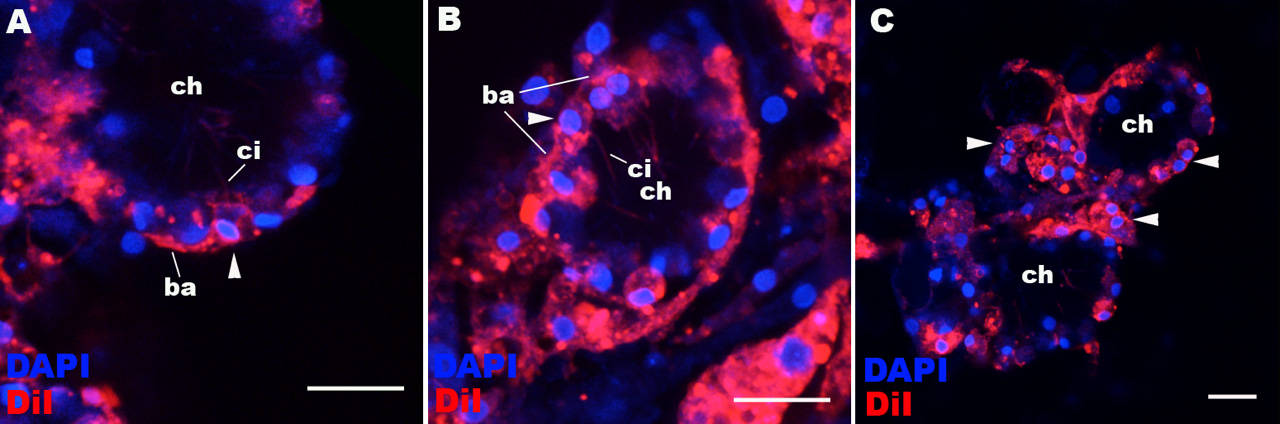

Supplement: Additional file 5: Figure S5 — Epithelial choanocytes can de-differentiate into archeocytes in A. queenslandica. Choanocytes in chambers (ch) in juveniles were labelled with CM-DiI as in Figure 3A, B and descendants of the labelled cells in juveniles one day later are shown. Some choanocytes are seen to extend basal processes (ba) laterally, engulfing neighboring choanocytes (arrowheads in A, B). Some cells appear multinucleated (arrowheads in C), presumably as a result of phagocytic or fusion events. Abbreviations: ch choanocyte chamber; ci cilium. Scale bar: 10 μm. [file 1741-7007-12-26-S5.jpeg]

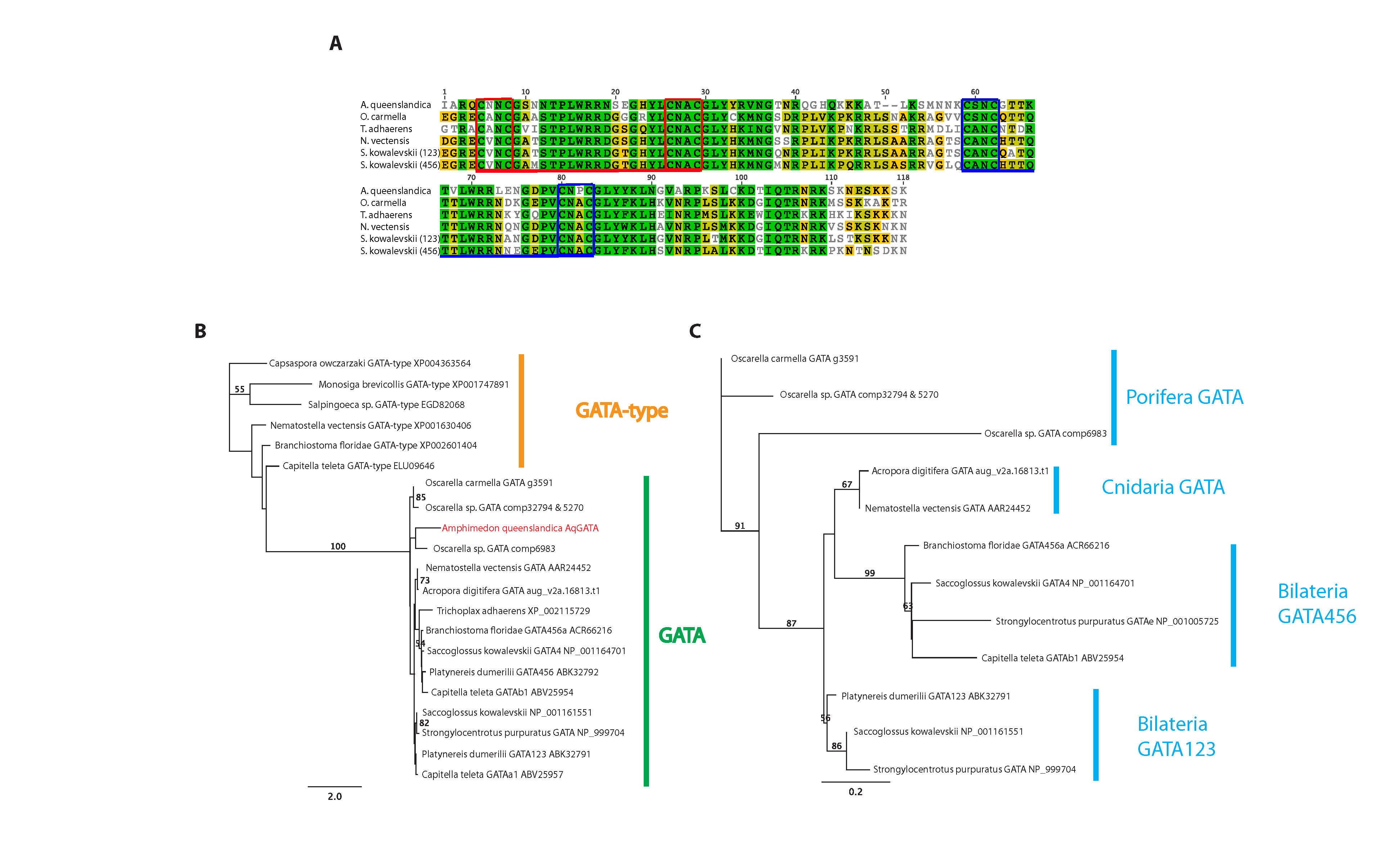

Supplement: Additional file 6: Figure S6 — GATA sequence alignment and phylogenetic analyses. A: Partial GATA protein multiple sequence alignment showing the amino acid sites used for phylogenetic analyses. GATA genes encode zinc-finger transcription factors that bind to the (T/A (GATA) A/G) cis-regulatory motif [45] and are characterised by the presence of two zinc-finger domains (N-terminal ‘N-finger’ and C-terminal ‘C-finger’). The alignment encompasses N-finger (underlined in red) and C-finger (underlined in blue) zinc-finger domains each encoding CXNC (boxed) X17CNXC (boxed). B: GATA maximum likelihood phylogeny rooted with GATA-type sequences. Amphimedon queenslandica sequence is highlighted in red. No clear GATA orthologues were identified outside Metazoa, and in the genome of the ctenophore Mnemiopsis leidyi[46]. C: Unrooted GATA maximum likelihood phylogeny. Sequence alignment and phylogenetic analyses were performed on the Geneious platform (v.5.1.7). Related sequences were retrieved via the protein BLAST search using the Amphimedon GATA-like sequence as queries, from GenBank at NCBI [47], Acropora digitifera genome (Version 1.1) portal at OIST [48], Mnemiopsis genome project portal at NIH [46] and Compagen at the University of Kiel (Oscarella sequences; [49]). Sequence IDs/accession numbers are shown with the name of each sequence in B and C. Peptide sequences were aligned with MUSCLE (v3.7) [50] (default settings), and ambiguous regions were manually removed. Phylogenetic trees were reconstructed using the maximum likelihood method implemented in the PhyML program [51]. The WAG substitution model [52] was selected assuming an estimated proportion of invariant sites and four gamma-distributed rate categories to account for rate heterogeneity across sites. The gamma shape parameter was estimated directly from the data. Reliability for internal branches of maximum likelihood trees was assessed using the bootstrapping method (100 bootstrap replicates). Support values are shown at each node except whe [file 1741-7007-12-26-S6.jpeg]
